# Supplementary material for: Predicting visual function by interpreting a neuronal wiring diagram
Source: Nature. 2024 Oct 2;634(8032):113–23. doi: 10.1038/s41586-024-07953-5 (PMC11446822; doi:10.1038/s41586-024-07953-5)
Supplement: Supplementary file 6 — CRF and ERF predictions for individual TmY4 and TmY9 cells. Analogous to Supplementary Data 3, but for TmY target types. Shown are the top four monosynaptic pathways, the strongest pathway passing through each of the top ten intermediary types (ranking from Extended Data Fig. 7), and the trisynaptic pathway Tm1–TmY–Dm3–TmY (see the section entitled Prediction of spatial normalization). [file 41586_2024_7953_MOESM6_ESM.zip › DataS4/TmY9q/720575940626993029.pdf]

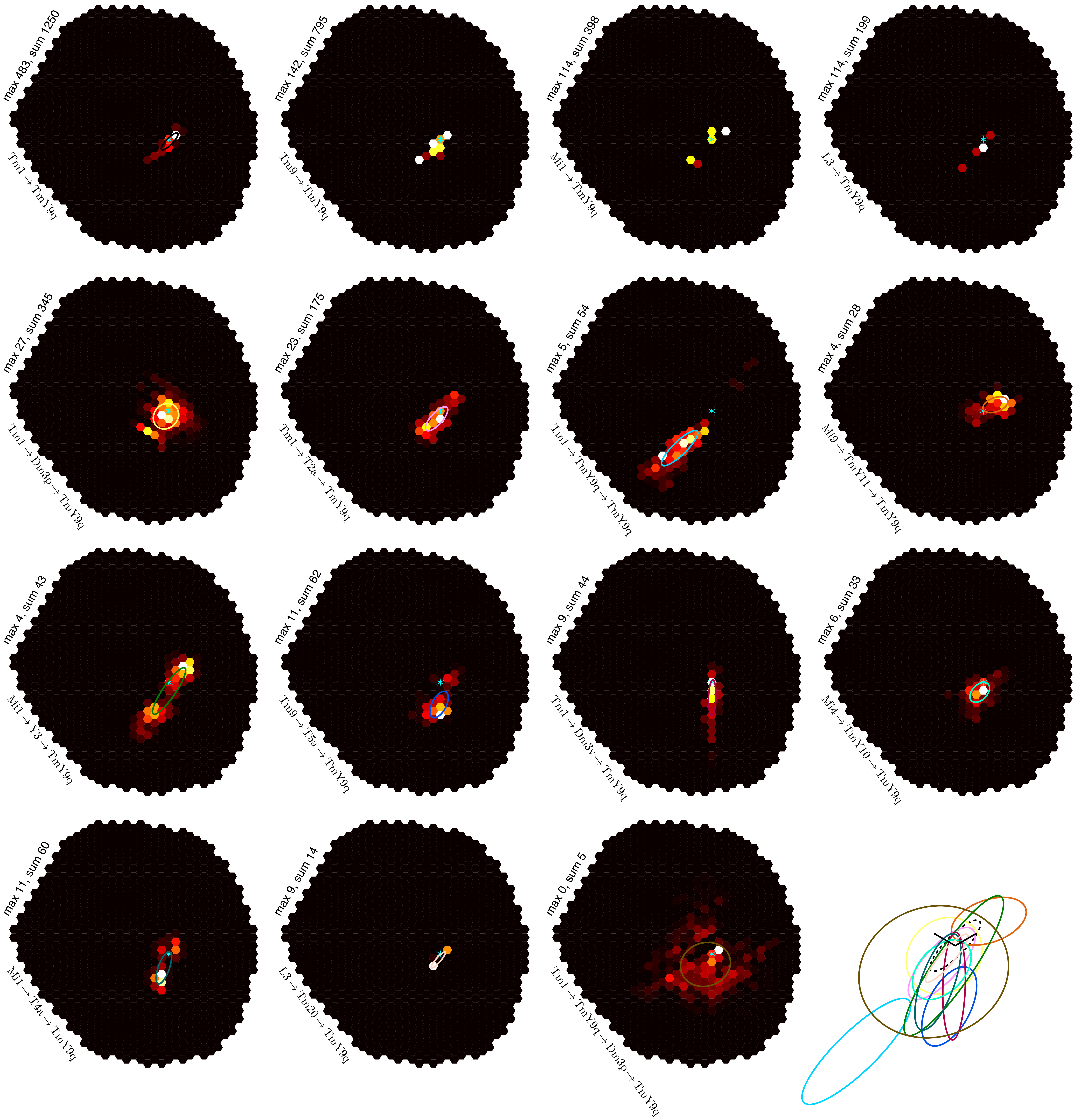
max 483, sum 1250
Tm1 → TmY9q
max 142, sum 795
Tm9 → TmY9q
max 114, sum 398
Mi1 → TmY9q
max 114, sum 199
L3 → TmY9q
max 27, sum 345
Tm1 → Dm3p → TmY9q
max 23, sum 175
Tm1 → T2a → TmY9q
max 5, sum 54
Tm1 → TmY9q → TmY9q
max 4, sum 28
Mi9 → TmY11 → TmY9q
max 4, sum 43
Mi1 → Y3 → TmY9q
max 11, sum 62
Tm9 → T5a → TmY9q
max 9, sum 44
Tm1 → Dm3v → TmY9q
max 6, sum 33
Mi4 → TmY10 → TmY9q
max 11, sum 60
Mi1 → T4a → TmY9q
max 9, sum 14
L3 → Tm20 → TmY9q
max 0, sum 5
Tm1 → TmY9q → Dm3p → TmY9q
